# Tipping the balance: NRF2’s dual role in ferroptotic fate

**DOI:** 10.1038/s41389-026-00630-4

**Published:** 2026-05-30

**Authors:** Izadora de Souza, Ana Beatriz da Silva Teixeira, Ancely Ferreira dos Santos, José Pedro Friedmann Angeli, Clarissa Ribeiro Reily Rocha

**Affiliations:** 1 Rudolf Virchow Zentrum (RVZ), Center for Integrative and Translational Bioimaging, University of Würzburg, Würzburg, Germany; 2 Department of Clinical and Experimental Oncology, Federal University of São Paulo (UNIFESP), São Paulo, Brazil; 3 Comprehensive Cancer Center Mainfranken, University Hospital Würzburg, Würzburg, Germany

**Keywords:** Cell death, Cancer metabolism, Cancer genetics

## Abstract

The NRF2 pathway has emerged as a central regulator of cellular redox homeostasis, coordinating the expression of a broad array of genes that protect cells from oxidative and electrophilic stress. In the context of cancer, NRF2 has been recognized as a key driver of chemoresistance, as its sustained activation enhances antioxidant defenses, detoxification pathways, and metabolic adaptation, thereby promoting tumor cell survival under therapeutic stress. Beyond its canonical role in redox regulation, NRF2 also orchestrates the expression of multiple genes involved in ferroptosis, a non-apoptotic, iron-dependent form of cell death that has recently gained attention as a promising strategy to overcome drug resistance. Mechanistically, NRF2 modulates ferroptosis through several interconnected pathways, including the regulation of glutathione biosynthesis, lipid metabolism, and iron homeostasis, yet its impact is highly context-dependent and can vary according to cell type and metabolic state. In this review, we provide an overview of the interplay between NRF2 and ferroptosis, tracing the historical development of this network and highlighting the pivotal roles of specific NRF2 targets in controlling ferroptotic susceptibility. Finally, we discuss how targeted modulation of NRF2 may influence ferroptosis, offering a potential avenue for the design of innovative therapies aimed at selectively eradicating resistant tumors.

## Introduction

**Fig. 1:**
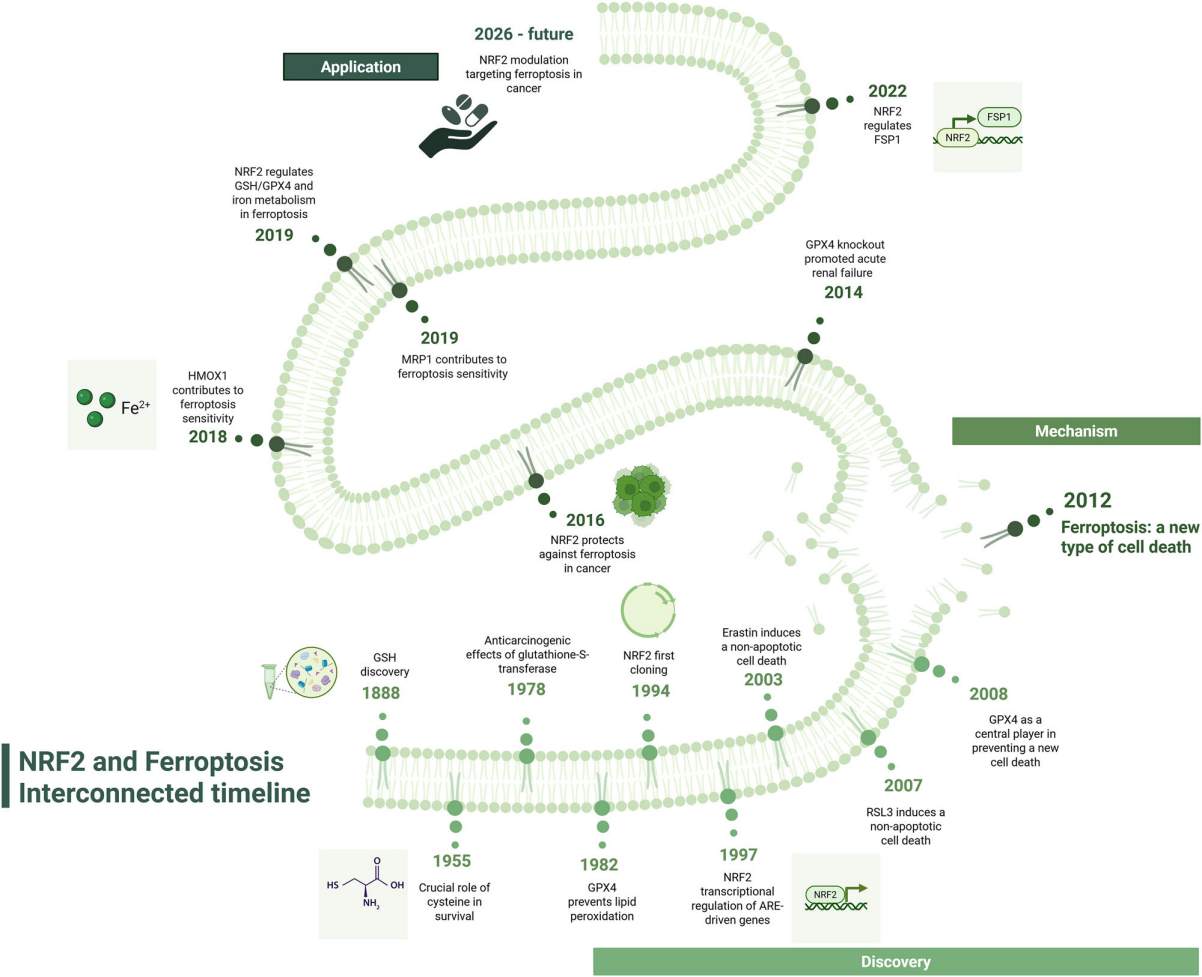
Interconnected historical outlines of NRF2 and ferroptosis. Timeline showing the evolution of the interplay between NRF2 and ferroptosis, including the past, present, and future developments. The discovery phase (1888–2012) began with the identification of GSH, progressed through the NRF2 first cloning, and concluded with Dixon et al. coining the term “ferroptosis” in 2012. The mechanism phase (2012–2023) was marked by rapid advancements, with research focusing on how NRF2 modulates ferroptosis. Looking ahead to the present and future (2026-future), numerous new applications are expected for targeting ferroptosis via NRF2 modulation in cancer. Created with BioRender.com.

Cancer cells typically exhibit higher levels of reactive oxygen species (ROS) than non-tumorigenic cells, largely as a result of mitochondrial dysfunction, metabolic reprogramming, and oncogenic signaling [1]. To cope with this heightened oxidative burden, tumor cells engage a range of antioxidant defense systems that limit oxidative damage and support survival [2]. The conceptual foundation of the antioxidant response was established in the 1970s, when several studies demonstrated that the anticarcinogenic effects of phenolic antioxidants stemmed from their ability to induce phase II detoxifying enzymes, including glutathione-S-transferase (GST) [3, 4]. These findings stimulated extensive investigation into the molecular pathways regulating glutathione (GSH) biosynthesis and function in cellular antioxidant defense [5] (Fig. 1). This body of work ultimately established GSH—a tripeptide composed of glycine, cysteine, and glutamic acid—as a central redox buffer and an essential cofactor for multiple antioxidant enzymes, including glutathione peroxidases, glutathione-S-transferases, and glyoxylases [5].

GSH is the most abundant intracellular peptide in human cells [6]. Although it was first identified in 1888, its pivotal role as a central regulator of cellular redox homeostasis was only fully appreciated in subsequent decades [6]. Early studies established GSH metabolism as a cornerstone of cell survival, linking cysteine availability and GSH depletion to oxidative cell death induced by acetaminophen toxicity and cystine deprivation [7–9]. In 1980, system xc⁻ was identified as a cystine-glutamate antiporter that imports cystine, which is then reduced intracellularly to cysteine, the rate-limiting substrate for GSH synthesis [10]. Importantly, in 1982, it was proposed a GSH-dependent peroxidase that protects biomembranes from lipid peroxidation, providing a mechanistic link between GSH metabolism and the suppression of oxidative membrane damage [11, 12].

During this period, growing evidence highlighted transcriptional control as a central mechanism underlying the GSH-dependent antioxidant response [4]. In 1994, nuclear factor (erythroid-derived 2)-like 2 (NRF2) was cloned and characterized as a NF-E2–related basic leucine zipper transcription factor [13]. Its physiological role, however, remained poorly understood until a study three years later demonstrated that the phenolic antioxidant butylated hydroxyanisole (BHA) induced the expression of GST and NAD(P)H:quinone oxidoreductase 1 (NQO1) in wild-type mice, but not in NRF2-deficient animals. Later, it was established that NRF2 mediates cytoprotective gene expression through direct binding to antioxidant response elements (AREs) in target promoters [14]. Since then, a series of groundbreaking investigations have revealed that NRF2 orchestrates the coordinated regulation of a diverse range of ARE-driven genes, such as *SLC7A11*/xCT, *GCLC, GCLM, GR, ABCC1*, directly involved in both the production and utilization of GSH [15–17]. Beyond GSH metabolism, NRF2 also regulates genes such as *HMOX1*, *FTL*, *FTH1* and *TXNRD1*, thereby providing protection against diverse intrinsic and extrinsic stressors [18–20]. Consequently, research focus shifted towards investigating the role of elevated NRF2 expression in oncogenesis, based on the premise that the inhibition of NRF2/GSH axis may be necessary to increase the sensitivity of cancer cells to chemotherapeutic agents [21, 22].

In parallel, a high-throughput screening designed to identify small molecules that selectively targeting HRAS^V12^ tumors led to the discovery of erastin (eradicator of RAS-transformed cells). Five years later, another compound, RAS-selective-lethal-3 (RSL3), was identified and shown to induce a non-apoptotic form of cell death dependent on oxidative stress accumulation and intracellular iron levels [23–25]. In 2008, Conrad and colleagues demonstrated that genetic disruption of GPX4 resulted in a type of cell death driven by lipid peroxidation, a process that could be attenuated by alpha-tocopherol [26]. Furthermore, they showed that overexpression of xCT protected cells from lipid peroxidation and, consequently, from this form non-apoptotic cell death [27].

Building on these discoveries, in 2012 Stockwell and colleagues coined the term ferroptosis to describe a regulated, iron-dependent form of cell death that is morphologically and biochemically different from apoptosis, necroptosis and unregulated necrosis [28]. They demonstrated that erastin triggers ferroptosis by inhibiting cystine uptake via system xc-, resulting in depletion of intracellular cysteine and GSH [29]. In parallel, GPX4 was identified as the direct molecular target of RSL3, consistent with earlier findings linking GPX4 disruption to lipid peroxidation-driven and cell death, establishing GPX4 as a central regulator of ferroptosis [26].

**Fig. 2:**
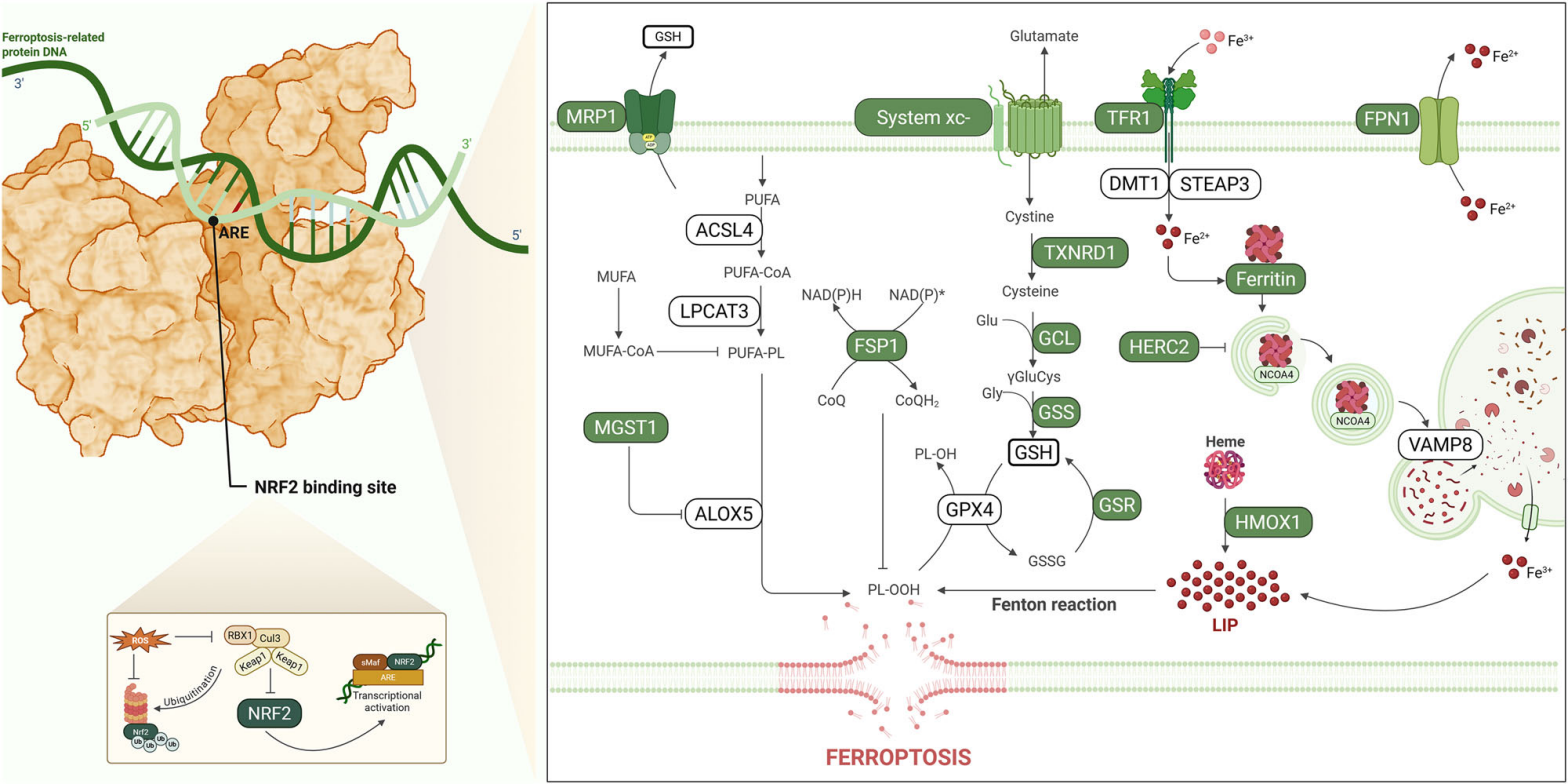
Detailed mechanisms of the ferroptosis cascade regulated by NRF2. Upon activation, NRF2 translocates to the nucleus to bind to the ARE region of various ferroptosis-related proteins. NRF2 controls the transcription of several proteins involved in the three primary pathways that mediate ferroptosis, including iron metabolism, redox, and lipid metabolism. Dysregulation of these proteins through NRF2 modulation can either inhibit or promote ferroptosis induction, depending on the context. Proteins highlighted in green are transcriptionally regulated by NRF2. FSP1, ferroptosis suppressor protein 1; FPN1, ferroportin; FSP1, ferroptosis suppressor protein 1; MGST1, microsomal glutathione S-transferase 1; HMOX-1, heme oxygenase 1; TFR1, transferrin receptor protein 1; MRP1, multidrug resistance-associated protein 1, TXNRD1, thioredoxin reductase 1; GCL, glutamate cysteine ligase; GSS, glutathione synthetase; GSR, glutathione reductase; HERC2, E3 ubiquitin protein ligase 2. Created with BioRender.com.

Subsequent studies have further elucidated the molecular mechanisms through which NRF2 modulates ferroptosis, revealing its involvement across multiple ferroptotic axes, including iron homeostasis, GSH metabolism and lipid metabolism [30] (Fig. 2). Notably, several NRF2-regulated proteins have been implicated not only in suppressing ferroptosis but also in enhancing susceptibility to this form of cell death, highlighting the context-dependent and dual nature of NRF2 signaling. In this Review, we summarize emerging evidence that positions NRF2 as a dynamic modulator of ferroptosis, whose function extends beyond cytoprotection to include pro-ferroptotic activities. Finally, we discuss how a deeper mechanistic understanding of NRF2-driven ferroptotic regulation may enable the rational design of therapeutic strategies that exploit ferroptosis for selective cancer cell elimination.

## Nrf2 and ferroptosis

NRF2 activity is tightly regulated at multiple levels to maintain redox homeostasis under physiological conditions [22]. Under basal conditions, NRF2 is retained in the cytoplasm by its negative regulator KEAP1, which promotes its ubiquitination and proteasomal degradation [30]. In response to oxidative stress or electrophilic insults, critical cysteine residues in KEAP1 are modified, allowing NRF2 stabilization, nuclear translocation, and activation of ARE-driven genes [30]. Additional layers of regulation involve post-translational modifications, interactions with other proteins such as p62/SQSTM1, and transcriptional control by oncogenic and tumor suppressor pathways [31, 32]. Notably, cancer cells often exhibit constitutive NRF2 activation, resulting from somatic mutations in *KEAP1* or *NFE2L2* that disrupt their interaction, as well as through oncogene-driven signaling and metabolic reprogramming that stabilize NRF2 [33]. This elevated NRF2 activity enhances the antioxidant capacity and survival of tumor cells, providing a selective advantage over non-transformed counterparts.

As described by Dixon and Stockwell, ferroptosis main regulation relies on three specific key features: the loss of lipid peroxide repair capacity, oxidation of polyunsaturated fatty acid (PUFA)-containing phospholipids, and the availability of redox-active iron [28]. NRF2 intersects with each of these regulatory axes, positioning it as a potential modulator of ferroptotic susceptibility. In that sense, through transcriptional control of genes involved in GSH biosynthesis and utilization, lipid metabolism, and iron homeostasis, NRF2 can profoundly influence the balance between lipid peroxidation and antioxidant defense. Consequently, NRF2 activation has been widely associated with ferroptosis resistance; however, emerging evidence indicates that NRF2 can also promote ferroptosis under specific cellular and metabolic contexts, underscoring its dual and context-dependent role. Elucidating how NRF2 integrates these ferroptotic pathways is therefore critical for understanding the molecular determinants of ferroptosis and for exploiting this form of cell death therapeutically, particularly in drug-resistant cancers (Fig. 2).

### NRF2 Anti-ferroptotic role

Shortly after the discovery of ferroptosis, GPX4 was established as a central regulator protecting cells from this form of death [34, 35]. GPX4 detoxifies membrane lipid hydroperoxides into non-toxic lipid alcohols through a catalytic reaction in which two molecules of GSH reduce a lipid hydroperoxide to glutathione disulfide (GSSG), water, and lipid alcohol [36]. Glutathione reductase (GSR) subsequently regenerates GSH from GSSG in an NADPH-dependent reaction, making GSH synthesis the rate-limiting step for GPX4 activity [11, 37]. Typically, the inhibition of system xc- or the suppression of GPX4 activity compromises cellular antioxidant capacity, leading to the accumulation of lipid peroxides and ultimately triggering ferroptosis [11, 38]. Notably, class I ferroptosis inducers, including erastin, IKE, and sulfasalazine, initiate ferroptosis by blocking cystine uptake through system xc- [39], whereas class II inducers, such as RSL3 and ML162, act primarily by targeting GPX4 activity [40]. In contrast, potent radical-trapping antioxidants (RTAs), such as ferrostatin-1 and liproxstatin-1, can prevent ferroptosis by scavenging lipid radicals and halting lipid peroxidation [28, 34].

Remarkably, the most recognized function of NRF2 is in preserving redox balance, mainly by facilitating the production and redox cycling of thiol-based antioxidant enzymes and GSH. Therefore, one of the main roles of NRF2 in preventing ferroptosis is through the regulation of the system xc- through the transcription of *SLC7A11* [15, 41]. NRF2 also governs the transcription of important enzymes and proteins involved in GSH synthesis and utilization, thereby contributing to ferroptosis suppression [17]. Thus, NRF2 activity led to ferroptosis resistance in NSCLC by inducing GCLM, SLC7A11, GSR, SRXN1, GPX2, and NQO1 gene expression [42]. In addition, a single-cell transcriptomic study revealed that in triple-negative breast cancer cells, NRF2 protects from ferroptosis through the activation of GSH-dependent peroxidases (MGST3 and PRDX6) [43]. ACTL6A was found to cooperate with NRF2 to regulate *GCLC* expression at the transcriptional level, facilitating GSH synthesis and inhibiting ferroptosis in gastric cancer cells [44]. Notably, disruption of NRF2 through pizotifen malate (PZM) treatment reduced GSH levels, enhanced ROS, and lipid peroxidation through the downregulation of key genes such as *GCLC, ME1*, and *G6PD* [45]. In summary, key strategies to sensitize resistant cancer cells to ferroptotic cell death involve targeting the availability of cysteine and synthesis of GSH, which are primarily coordinated by NRF2.

The molecular mechanisms governing ferroptosis have grown far beyond the initial GPX4 regulatory pathway, uncovering a complex and interconnected regulatory network. It has been described three distinct GPX4-independent mechanisms that function to suppress ferroptosis: ferroptosis suppressor protein 1 (FSP1)/CoQ10, dihydroorotate dehydrogenase (DHODH), and GTP cyclohydrolase 1 (GCH1)/tetrahydrobiopterin (BH4). FSP1 and DHODH are involved in reducing ubiquinone (CoQ) to ubiquinol (CoQH2) on the plasma and inner mitochondrial membrane, respectively [46–48]. This process allows CoQH2 to serve as an RTA, effectively neutralizing lipid peroxyl radicals. Accordingly, GCH1 expression suppresses ferroptosis by generating lipophilic antioxidant BH4, which functions analogously to CoQ10 in preventing lipid peroxidation and depleting the PUFA-PLs [49, 50]. Of note, class III FINs have been identified to deplete GPX4 and CoQ10 through the SQS-mevalonate pathway, exemplified by FIN56 [51].

Gan et al. identified FSP1 as a direct NRF2 target whose upregulation in KEAP1-mutant cancers promotes ferroptosis resistance and poor survival, whereas inhibition of the CoQ–FSP1 axis restores ferroptotic sensitivity [47]. Recent findings have shown that high expression of NRF2 was correlated with higher levels of FSP1 in pancreatic ductal adenocarcinoma (PDAC) [50]. Moreover, NRF2 downregulation in combination with FSP1 inhibitors significantly increased the sensitivity to ferroptosis in cancer cells [42]. In summary, these findings place CoQ–FSP1 as a crucial downstream effector in the NRF2 pathway to suppress ferroptosis in many cancer cell types.

Lipid peroxidation is an intricate chain chemical reaction involving free radicals or non-radical-driven processes initiated by the interaction of ROS with polyunsaturated fatty acids (PUFAs) [34]. This interaction leads to the generation of hydroperoxides and lipid peroxyl radicals, occurring in distinct phases: initiation, propagation, and termination, as extensively reviewed by Yin et al. [52]. It is noteworthy that free PUFAs do not drive ferroptosis; rather, the activities of ACSL4 and LPCAT3 are essential for activating and incorporating PUFAs, such as arachidonic acid, into membrane phospholipids, enabling their subsequent peroxidation and lethality [40, 53, 54].

On the other hand, enzymes involved in modifying phospholipids, such as MBOAT1 and MBOAT2, inhibit ferroptosis by altering the cellular phospholipid profile in a GPX4 and FSP1-independent manner [55]. Recent studies have shed light on the previously unrecognized anti-ferroptotic activity of 7-dehydrocholesterol (7-DHC), a cholesterol precursor. This molecule protects phospholipids from autoxidation and subsequent fragmentation, thus suppressing ferroptosis [56, 57]. Lipoxygenases (LOXs) also play an important role in modulating ferroptosis by contributing to membrane destabilization and the oxidation of PUFAs, ultimately releasing harmful aldehydes [58, 59].

Although relatively few studies have addressed it, NRF2 is emerging as a key regulator of lipid metabolism. Kuang et al. unveiled the role of MGST1 in preventing ferroptotic cell death through binding and subsequently inactivating ALOX5, thereby decreasing the production of oxidized PUFAs [60]. Interestingly, it was demonstrated that MGST1 is positively correlated with NRF2 expression in pancreatic tumors, which leads to a poor prognosis. Luciferase reporter gene assays confirmed that MGST1 is a target gene of NRF2. Of note, NRF2 or MGST1 deficiency sensitized pancreatic cancer cell xenografts to IKE-induced ferroptosis [60]. In parallel, NRF2 activation suppresses ferroptosis by remodeling lipid metabolism via PIR–PLA2G4A in colorectal cancer and the SCD1–SLC7A11 axis in lung adenocarcinoma [61, 62]. Overall, NRF2 modulation may be explored to suppress lipid peroxidation through the regulation of lipid metabolism.

Excessive intracellular redox-active iron can drive ROS production via the Fenton reaction, promoting lipid peroxidation and ferroptosis [63]. Cellular iron homeostasis is therefore tightly controlled through coordinated regulation of iron uptake, transport, storage, and metabolism [64, 65]. Once within the cell, iron can be exported by ferroportin or sequestered by ferritin, with NCOA4-mediated ferritinophagy releasing ferritin-bound iron into the labile iron pool (LIP) [66, 67]. Elevated LIP fuels lipid peroxidation and ROS generation, triggering ferroptotic cascades, and HMOX1 contributes to this process by degrading heme to release Fe²⁺, although its activity also produces biliverdin and induces ferritin expression, providing a cytoprotective effect [68, 69].

NRF2 orchestrates the transcription of multiple genes involved in iron homeostasis, including *FTL*, *FTH1*, *SLC40A1*, and *HMOX1*, underscoring its central role in maintaining cellular iron balance [18, 70]. In KEAP1-mutant cells resistant to ferroptosis, NRF2 activation markedly elevates *FTH1* and *FTL* levels [42]. Clinically, patients with acute myeloid leukemia (AML) exhibit increased NRF2 and *FTH1* expression, correlating with poor prognosis, while pharmacological inhibition of NRF2 using ML385 reduces *FTH1* levels, elevates the labile iron pool, and enhances venetoclax-induced lipid peroxidation [71]. Similarly, in macrophages, RSL3 promotes ferroportin expression via NRF2 nuclear accumulation and BACH1 downregulation, thereby decreasing lipid peroxidation [72].

A recent study demonstrated that NRF2 directly drives *HERC2* transcription, which, in the presence of free iron, binds NCOA4 and inhibits ferritin degradation [31]. NRF2 depletion also reduces VAMP8 levels, impairing the final step of ferritinophagy and causing accumulation of apoferritin and NCOA4 in autophagosomes, thereby increasing the labile iron pool (LIP) and sensitizing cancer cells to ferroptosis [73]. Thus, while NRF2 can promote macroautophagy via VAMP8, it simultaneously suppresses ferritinophagy through NCOA4 inhibition [31]. It has been shown that erastin induces a compensatory NRF2 response that protects cancer cells during ferroptosis, but this adaptive increase is abrogated by CISD2 depletion or NCOA4 knockdown, highlighting the intertwined roles of CISD2, ferritinophagy, and NRF2/HMOX1 regulation [74].

### NRF2 Pro-ferroptotic role

In addition to its well-characterized role in protecting cells from oxidative stress, NRF2 can paradoxically promote ferroptosis under certain conditions, with this pro-ferroptotic effect largely mediated through the transcriptional induction of HMOX1 [18]. Despite the antioxidant and anti-inflammatory properties associated with HMOX1, recent studies have demonstrated that increased levels of this enzyme can promote the accumulation of Fe^2+^, impairing iron homeostasis and triggering ferroptosis, counterbalancing the protective effect of NRF2 [75].

Several studies have highlighted *HMOX1* induction as a key driver of ferroptosis in cancer cells. Withaferin-A (WA) promotes ferroptosis in high-risk neuroblastoma via KEAP1 inhibition, increasing *HMOX1* while decreasing GPX4 [68]. Zoledronic acid triggers ferroptosis in osteosarcoma cells by upregulating HMOX1 and reducing CoQ10, whereas EF24, a curcumin analog, induces ferroptosis through HMOX1 activation [76, 77]. BAY 11-7085, an IκBα inhibitor, similarly induces ferroptosis via *HMOX1*-dependent iron accumulation independently of NF-κB [78]. In hepatocellular carcinoma, GSK-J4 combined with donafenib causes ferroptotic cell death associated with increased NRF2 and *HMOX1*, which is driven by enhanced promoter–enhancer interactions and is suppressed by HMOX1 inhibition [79]. Flavonoids such as quercetin and 4,4′-dimethoxychalcone (DMC) induce ferroptosis through KEAP1 degradation and *HMOX1* upregulation, increasing labile iron and ROS in resistant tumor cells like A549 and 786-O [80, 81]. Similarly, isoliquiritigenin (ISL) inhibits gallbladder cancer proliferation and promotes ferroptosis by upregulating *HMOX1* while downregulating GPX4 [82].

Overall, accumulating evidence suggests that HMOX1 can both facilitate and limit ferroptosis, with its intracellular activity level likely determining the outcome. This resembles a hormetic response, in which moderate HMOX1 induction confers protection, whereas excessive activation becomes detrimental by increasing free iron beyond ferritin’s buffering capacity. However, the precise mechanisms by which HMOX1 upregulation, alongside other cytoprotective NRF2 targets, triggers ferroptosis remain unclear.

Notably, cancer cells display a heightened demand for iron—a phenomenon termed “iron addiction”—rendering them particularly susceptible to ferroptosis [83]. Class IV ferroptosis inducers (FINs), including withaferin A, hemin, and ferrous ammonium sulfate ((NH₄)₂Fe(SO₄)₂), can initiate lipid peroxidation by increasing labile iron or promoting its oxidation [68]. Hemin also degrades BACH1 via the proteasome, enhancing NRF2 activity and *HMOX1* expression to further drive ferroptosis [68, 84] while (NH₄)₂Fe(SO₄)₂ promotes iron accumulation in neuroblastoma cells, triggering ferroptotic death. Collectively, these compounds represent promising therapeutic strategies for ferroptosis-driven diseases, warranting further investigation.

In addition to *HMOX1*, another NRF2-regulated factor with pro-ferroptotic potential is *ABCC1*, which encodes the multidrug resistance protein 1 (MRP1) [85, 86]. It is described that while MRP1 promotes the efflux of GSH-drug conjugates, contributing to drug resistance, it can also export GSH itself, leading to intracellular GSH depletion under certain conditions [85, 87]. Although MRP1-mediated export of 4-HNE conjugates contributes to anti-ferroptotic effects, its inhibition was shown to preserve intracellular GSH, attenuate ferroptosis, and reduce myocardial infarct size in vivo following ischemia-reperfusion [88, 89]. Dixon and colleagues reported that high MRP1 levels confer resistance to pro-apoptotic chemotherapeutics while simultaneously sensitizing cells to ferroptotic inducers such as erastin2, RSL3, ML162, and BSO [75]. Similarly, glioblastoma cells with elevated NRF2 evade TMZ-induced cell death by increasing GSH-conjugate efflux via MRP1, yet this same mechanism renders them more susceptible to ferroptosis through GSH depletion [90].

These findings suggest that although NRF2 upregulation boosts intracellular GSH levels, its ability to prevent ferroptosis is limited, partly because NRF2 also upregulates MRP1 [91, 92]. The reasons why NRF2’s pro-ferroptotic effects can override its antioxidant actions on other targets remain unclear and warrant further investigation. Notably, ABCC1 has been identified as a ferroptosis-related gene in several types of cancer, and its higher expression was associated with poor overall survival in nasopharyngeal carcinoma, uveal melanoma, bladder cancer, and glioblastoma [93–95]. Importantly, these findings highlight a potential strategy to enhance chemotherapeutic efficacy in patients with *ABCC1*-upregulated tumors by exploiting ferroptosis induction.

## Targeting nrf2 and ferroptosis in clinical practice

Gene signature, proteomic, and lineage-based analyses have revealed that cancer cells undergoing epithelial-mesenchymal transition and those resistant to apoptosis are particularly vulnerable to ferroptosis [96]. This selective susceptibility makes ferroptosis an attractive therapeutic target, as it offers a means to eliminate tumor cells that evade conventional treatments. Indeed, ferroptotic interventions have been shown to enhance the efficacy of chemotherapeutics such as temozolomide, cisplatin, haloperidol, and doxorubicin across multiple tumor types [90, 97–99]. Yet, despite this promise, no ferroptosis inducers have reached clinical application, largely due to challenges in drug bioavailability and tumor-specific targeting.

For instance, erastin is not suitable for in vivo use due to its low solubility and metabolic instability. Recent efforts in drug development and repurposing offer solutions: erastin derivatives, such as imidazole ketone erastin, with improved pharmacokinetic properties, effectively inhibited tumor growth in fibrosarcoma and lymphoma xenograft models [100]. In turn, RSL3 is not suitable for use in vivo because it has many off-target effects on other selenoproteins. Similarly, molecules that target allosteric sites on other GPX4 cysteine residues, such as LOC1886, require high concentrations to inhibit GPX4 and may not yet be suitable for in vivo use [101]. The same scenario applies to FIN56, which is a more selective analog of the compound CIL56 (CIL: caspase-3/7-independent lethal) but also displays metabolic instability and exhibits off-target effects on squalene synthesis [51].

To enhance the in vivo efficacy of ferroptosis inducers, researchers are exploring advanced delivery strategies, with nanomedicine at the forefront due to its unique properties [102]. Ultrasmall poly(ethylene glycol) (PEG)-coated silica nanoparticles were the first to induce ferroptosis and reduce tumor growth by delivering iron directly into cells, and early clinical trials suggest they are safe for human use [103]. Other platforms, such as FePt-NP2, boost ROS production in combination with cisplatin [104], while dynamic nanoparticles like BNP@R and injectable hydrogels such as RTFG@SA allow targeted delivery of RSL3 [105]. Despite these promising approaches, further preclinical work is needed to optimize targeting, biocompatibility, biodegradability, and immunogenicity [106]. Combining nanomedicine with additional pro-ferroptotic strategies may yield more effective cancer therapies.

### Clinical challenges in modulating ferroptosis through direct NRF2 modulation

As observed in many cases, elevated NRF2 leads to ferroptosis resistance in tumors, making its inhibition a promising therapeutic strategy. Several compounds, including the natural product brusatol, have been identified as NRF2 inhibitors; however, they lack target specificity limits their utility [107]. More recently, VVD-065, a first-in-class NRF2 inhibitor, was shown to covalently target KEAP1 Cys151, promoting NRF2 degradation and thereby suppressing tumor growth while enhancing chemo- and radiosensitivity, supporting an ongoing Phase I clinical trial (NCT05954312) [108]. A similar limitation applies to pizotifen malate (PZM), an FDA-approved drug for esophageal squamous cell carcinoma, which — despite being reported to suppress NRF2 transcriptional activity by disrupting ARE binding — also functions as a serotonin receptor antagonist and therefore cannot be considered a bona fide NRF2 inhibitor [45]. Although multiple approaches have been explored to identify more selective NRF2 inhibitors, such as PROTACs, small molecule glue-like compounds, and fragment-based screening strategies [109–111], the absence of a highly specific NRF2 inhibitor currently precludes the clinical implementation of this strategy.

In contrast to the challenges associated with NRF2 inhibition in cancer, NRF2 activation has shown considerable clinical progress in non-neoplastic diseases. Indeed, nearly 100 clinical trials currently investigate NRF2 activators to treat non-neoplastic diseases can currently be found on Clinicaltrials.gov. Recently, Sotorasib and adagrasib, KRAS inhibitor anti-cancer drugs, were found to activate NRF2 via KEAP1 cysteine modification, inducing NRF2-dependent transcription [112]. In fact, among clinical applications targeting NRF2 and ferroptosis, neurodegenerative disorders represent one of the most advanced areas, as NRF2 activation and ferroptosis inhibition converge on the shared goal of limiting oxidative stress in these diseases. This association is exemplified by omaveloxolone, the first drug approved for Friedreich’s ataxia, which improves neurological outcomes through NRF2 activation and has also been shown to protect against ferroptosis-mediated cell death [113]. Thus, the clinical success of omaveloxolone in neurological disease could be attributed, at least in part, to NRF2-mediated inhibition of ferroptosis, underscoring the significance of exploring this relationship to enhance the effectiveness of treatments in non-neoplastic diseases.

While the concept of NRF2 inhibition or activation as a crucial mechanism to induce or prevent ferroptosis is widely recognized, the role of NRF2 appears to be context-dependent, (Table 1). NRF2 activation is generally protective in neurodegenerative settings, however sustained or excessive activation may upregulate pro-ferroptotic targets such as HMOX1 and MRP1, potentially undermining therapeutic benefit. Conversely, NRF2 inhibition may also blunt ferroptosis by reducing the expression of these same targets. Supporting this complexity, sulforaphane-induced NRF2 activation in erythroid progenitors increases both pro- and anti-ferroptotic genes, including *HMOX1* and *NQO1*, in a dose-dependent manner [114]. Thus, whether NRF2 activation can be safely leveraged therapeutically, such as in sickle cell disease, without triggering ferroptosis remains uncertain.

**Table 1:** NRF2 target genes involved in ferroptosis modulation.

Cancer	NRF2 status	NRF2-target association	Experimental Model	Ferroptosis impact	Clinical correlation	References
Breast cancer	High NRF2 levels in breast cancer are associated with tumor progression and poor prognosis.	NRF2-dependent regulation of **MGST3 and PRDX6**	Breast cancer cells with p53 mutations (humans - MDA-MB- 231, HCC1395, BT549, HCC38,	Resistance	The overall survival of breast cancer patients with a high expression of six antioxidant genes, including *PRDX6* and *MGST3*, was significantly worse.	[43, 141]
			HCC1395- and primary murine- P172CC and P245CC)			
Glioma	Human GBMs have higher NRF2 levels compared to non-cancerous tissue, which is associated with poorer clinical outcomes.	Activation of the NRF2-KEAP1 signaling upregulates **xCT**	Glioma cell lines (F98 and U87)	Resistance	.	[41]
		**ABCC1/MRP1** upregulation by NRF2	Glioblastoma cell lines (U251MG and T98G)	Sensitivity	NRF2 correlates with *ABCC1* in glioma patients’ tumor tissues, linked to aggressiveness, drug resistance, and lower survival rates	[90]
Lung cancer	Lung cancer is the tumor type with the most NRF2 and KEAP1 mutant cases.	NRF2 is capable of elevating the expression of ferroptosis-targets (**GCLM, SLC7A11, GSR, SRXN1, GPX2, NQO1, FTH1, and FTL**), with **FSP1** emerging as the most significant contributor	KEAP1 mutant NSCLC cells lines (A549, NCI-H460, NCI-H2009, NCI-H1437, NCI-H322 and NCIH23)	Resistance	FSP1 upregulation was observed in LUAD patients with KEAP1 mutations, correlating with enhanced NRF2 pathway regulation.	[142, 143]
		NRF2/**HMOX1** axis	Human NSCLC (HCC827, H1299, H23, H820, H1975, PC9, H1573, H1793, H460, and A549)	Sensitivity		[142]
		**MRP1** upregulation by NRF2	A549, H1299	Sensitivity		[75]
Osteosarcoma	High levels of NRF2 are associated with a poor outcome and disease-free survival in osteosarcoma.	**MRP1** upregulation by NRF2	U2-OS	Sensitivity		[75, 144]
Gastric cancer	Patient survival is significantly poorer in gastric tumors harboring NRF2 activation.	NRF2 upregulates ***GCLC*** as a cotranscription factor with ACTL6A	Gastric cancer cell lines (SNU638 SNU216 and SNU668)	Resistance	GCLC expression was significantly positively correlated with ACTL6A, and high levels of both were associated with poor overall survival.	[44, 142]
Pancreatic Cancer	KEAP1/NRF2 mutations are uncommon; however, NRF2 is upregulated in over 93% of the cases.	***MGST1*** is a target gene of NRF2	Human pancreatic ductal adenocarcinoma cell lines (CFPAC1 and PANC2.03)	Resistance	MGST1 is positively correlated with NRF2 expression in pancreatic tumors, which leads to a poor prognosis.	[60, 145]
		**FSP1** is upregulated because of NRF2 pathway activation downstream of KRAS	Pancreatic organoids derived from the LsL-KRASG12D expressing mouse model	Resistance	Upregulation of FSP1 has been observed in pancreatic ductal adenocarcinoma tissues. This upregulation correlates with NRF2 expression in PDAC patient datasets.	[145, 146]
Cervical cancer	Mutations in R34, the most frequently mutated residue of NRF2 are found in cervical tumors.	NRF2/**HMOX1** axis	Cervical cancer cell lines (HeLa and SiHa)	Sensitivity	There is a correlation between high HMOX1 expression and lymph node metastasis, advanced clinical stage, and poor prognosis.	[142, 147]
Esophageal cancer	The upregulation of NRF2 was found to have a negative correlation with patient prognosis and to promote tumor proliferation in ESCC.	Regulatory transcription of ***GPX4, GCLC, ME1*** and ***G6PD*** via NRF2	Esophageal squamous cell carcinoma (ESCC) PDX model	Resistance		[45]
Ovarian cancer	Epithelial ovarian cancer has been demonstrated to exhibit elevated levels of NRF2.	NRF2 transcribes ***HERC2*** and leads to **NCOA4** inhibition	Ovarian cancer cells (SKOV-3, CAOV-3, MES-OV, SW-626, OVCAR-3, and OVCAR-8) and preclinical models, including ovarian tumor spheroids, xenografts, and patient-derived tumors	Resistance	NRF2 levels correlate with HERC2 and VAMP8 in human ovarian cancer tissues, as well as ferroptosis resistance in a panel of cell lines	[73]
Acute myeloid leukemia (AML)	NRF2 expression was significantly elevated in AML patients, being correlated with an inferior survival rate.	NRF2 induces **FTH1** expression	Acute myeloid leukemia cell lines (MV411, MOLM13, HL60, THP1, and NB4)	Resistance	Patients with acute myeloid leukemia (AML) display higher expression of NRF2 and FTH1, leading to a dismal prognosis.	[71]

Thus, although NRF2’s dual role in ferroptosis suggests that both its inhibition and activation could be therapeutically exploited, targeting specific NRF2 downstream effectors offers a more precise and clinically viable approach. Accordingly, the following section focuses on the most advanced modulators of NRF2-regulated targets that may enhance ferroptosis sensitivity in cancer.

### Inhibitors of anti-ferroptotic NRF2 targets

NRF2 downstream targets are central regulator of redox balance and ferroptosis sensitivity, making them attractive therapeutic nodes. Key pathways include cystine import, glutathione synthesis, quinone detoxification, lipid radical scavenging, and iron storage. Among these, inhibition of xCT, a canonical NRF2 target, is one of the most established strategies to induce ferroptosis. Although sulfasalazine, has been explored as a repurposed ferroptosis inducer due to its ability to block system xc⁻ in vitro, the concentrations required exceed those achievable in patients, limiting its clinical utility [115]. Similarly, while sorafenib was initially reported to induce ferroptosis via system xc⁻ inhibition, subsequent studies have shown that it does not act as a bona fide system xc⁻ inhibitor, in contrast to compounds such as erastin [116–118].

The cysteine-reactive ligand EN25 selectively targets an allosteric cysteine (C114) on GCLM, impairing GCL activity, depleting intracellular GSH, and compromising cell survival, with ARID1A-deficient cancer cells showing heightened sensitivity [119]. Similarly, direct inhibition of GCLC using compounds like L-buthionine sulfoximine (BSO) leads to GSH depletion and induction of ferroptosis [120], though clinical translation is limited by the lack of tumor specificity and potential toxicity of those drugs. Emerging evidence highlights post-translational modifications (PTMs) as an alternative regulatory layer: SIRT2 stabilizes GCLC via desuccinylation during ferroptosis, and its inhibition with SirReal2 enhances ferroptotic sensitivity, especially alongside RSL3, suggesting PTM-targeted approaches may help overcome ferroptosis resistance [121].

Dicoumarol (DIC), an FDA-approved NQO1 inhibitor, has emerged as a potential therapeutic agent for ferroptosis induction. In hepatocellular carcinoma models, DIC overcomes intrinsic resistance of KEAP1-deficient cells to class II ferroptosis inducers, highlighting NQO1’s role in NRF2-driven ferroptotic escape [122, 123]. For lipid peroxyl radical scavenging, FSP1 inhibitors have been widely pivotal: first-generation iFSP1 is human-specific, limiting preclinical use, whereas next-generation, species-independent inhibitors like viFSP1 effectively target FSP1’s NAD(P)H-binding pocket and show therapeutic promise when combined with sorafenib via biomimetic nanoparticles [124]. Mechanistic studies have further clarified their binding modes, providing a structural framework for rational drug design [125, 126].

In iron metabolism, salinomycin and its derivatives selectively target breast cancer stem cells by promoting lysosomal iron sequestration, triggering ferritin degradation, lysosomal iron overload, ROS generation, and ferroptosis [127]. Early clinical evaluation reported partial regression of metastatic disease with a favorable safety profile, though long-term integration remains uncertain [128]. Similarly, artesunate (ART) induces ferroptosis by promoting ferritinophagy via upregulation of NCOA4, increasing intracellular Fe²⁺ levels, lipid peroxidation, and tumor cell death. In osteosarcoma xenografts, ART suppressed tumor growth and, being already approved for malaria with a good safety profile, represents a promising candidate for repurposing in ferroptosis-based cancer therapy [129, 130].

### Inducers of pro-ferroptotic NRF2 targets

While most strategies focus on suppressing NRF2-driven anti-ferroptotic defenses, activation of specific NRF2-targets can also promote ferroptosis. Among these, targets involved in heme metabolism and glutathione efflux act as context-dependent pro-ferroptotic effectors. Hemin, the oxidative form of heme, promotes proteasomal degradation of the transcriptional repressor BACH1, upregulates HMOX1, and increases heme catabolism, iron release, and oxidative stress, sensitizing cells to ferroptosis [131]. Likewise, the HMOX1-inducing compound HPP-4382 inhibits BACH1 without disrupting ROS or glutathione levels [132]. FDA-approved since 1983 for porphyria, hemin is being explored for repurposing in other diseases, though its pharmacological profile and potential adverse effects require further study [133]. Importantly, the relatively small number of known pharmacological modulators of HMOX1 underscores a significant gap in the field and highlights the need for the development of more potent and selective HMOX1-targeting agents.

Beyond heme metabolism, NRF2-linked glutathione transport represents another axis with pro-ferroptotic potential. MRP1-overexpressing drug-resistant cancer cells can be selectively killed by flavonoids that induce intracellular glutathione depletion through MRP1-mediated efflux, triggering apoptosis via collateral sensitivity [134]. Phenothiazine maleates were shown to enhance the transport of fluorescent MRP1 substrates [135]. Small molecules such as the human immunodeficiency virus type 1 (HIV-1) protease inhibitors indinavir, nelfinavir, and ritonavir stimulated GSH efflux in human astrocytes and intestinal epithelial cells by inducing MRP1 activity [136, 137]. More recently, two novel classes of MRP1 activators, pyrrolopyrimidine derivatives and purine analogs, have been characterized [138]. Lower-molecular weight derivatives were found to stimulate MRP1-mediated transport of established substrates such as calcein AM and daunorubicin.

Although NRF2 modulators show promise, clinical application remains distant. Another avenue is using NRF2 and its targets as tumor biomarkers to identify cells susceptible to ferroptosis, optimizing therapeutic windows and minimizing toxicity. For example, an NRF2 pathway gene signature of 28 genes has been linked to cisplatin/vinorelbine response in NSCLC [139]. Developing comparable signatures could predict tumor response to ferroptosis inducers, guide dosing, and tailor therapy based on NRF2 status and target expression, taking into account tumor context and clinical relevance (Table 1).

## Concluding remarks

**Fig. 3:**
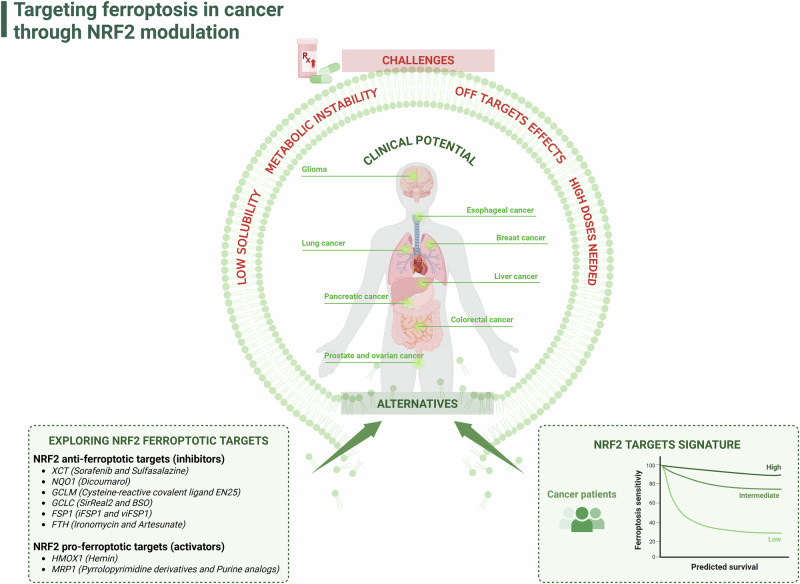
Targeting ferroptosis in cancer through NRF2 modulation. This schematic overview explores how NRF2’s dual role in ferroptosis could be leveraged to advance ferroptotic treatments in clinical settings. The clinical application of ferroptosis inducers faces several obstacles, such as the compounds’ metabolic instability, low solubility, the need for high doses to achieve sensitization, and off-target effects, among others. In this context, NRF2 modulation could serve as an alternative approach to bring ferroptosis treatment closer to clinical application. These alternatives include exploring NRF2-targets inhibitors (as detailed on the left box) and conducting NRF2 target signatures for various cancer types to assess potential sensitivity to ferroptosis in each case (right box). Created with BioRender.com.

Given its pivotal and context-dependent role in the regulation of ferroptosis, modulation of NRF2 has emerged as a particularly promising strategy for translating ferroptosis-related therapies into clinical practice. Accumulating evidence indicates that NRF2 can both promote and impair this form of cell death, underscoring its dual and complex functions. As highlighted in a recent review by Nakamura and Conrad, “despite remaining uncertainties and important considerations, the question is not if, but when the first ferroptosis-based cancer therapy will begin.” [140]. In this context, fully elucidating the role of NRF2 in ferroptosis could accelerate this timeline. Whether through the use of modulators targeting NRF2-regulated pathways to induce ferroptosis in cancer cells, or by leveraging NRF2 activity as a biomarker to identify ferroptosis-susceptible tumors, NRF2 may ultimately serve as a critical bridge to translate ferroptosis into clinical application (Fig. 3).

## References

[1] Trachootham D, Alexandre J, Huang P. Targeting cancer cells by ROS-mediated mechanisms: a radical therapeutic approach? Nat Rev Drug Discov. 2009;8:579–91. 10.1038/nrd2803. 19478820 10.1038/nrd2803

[2] Cheung EC, Vousden KH. The role of ROS in tumour development and progression. Nat Rev Cancer. 2022;22:280–97. 10.1038/s41568-021-00435-0. 35102280 10.1038/s41568-021-00435-0

[3] Dodson M, De La Vega MR, Cholanians AB, Schmidlin CJ, Chapman E, Zhang DD. Modulating NRF2 in disease: timing is everything. Annu Rev Pharm Toxicol. 2019;59:555–75. 10.1146/annurev-pharmtox-010818-021856. 10.1146/annurev-pharmtox-010818-021856 PMC6538038 30256716

[4] Benson AM, Batzinger RP, Ou SY, Bueding E, Cha YN, Talalay P. Elevation of hepatic glutathione S-transferase activities and protection against mutagenic metabolites of benzo(a)pyrene by dietary antioxidants. Cancer Res. 1978;38:4486–95. 363262

[5] Lu SC. Regulation of glutathione synthesis. Mol Asp Med. 2009;30:42–59. 10.1016/j.mam.2008.05.005. 10.1016/j.mam.2008.05.005 PMC2704241 18601945

[6] Alanazi AM, Mostafa GAE, Al-Badr AA. Glutathione. in Profiles of drug substances, excipients and related methodology. Elsevier; 2015. p. 43–158. 10.1016/bs.podrm.2015.02.001. 10.1016/bs.podrm.2015.02.001 26051685

[7] Mitchell JR, Jollow DJ, Potter WZ, Davis DC, Gillette JR, Brodie BB. Acetaminophen-induced hepatic necrosis. I. Role of drug metabolism. J Pharm Exp Ther. 1973;187:185–94. 4746326

[8] Eagle H. Nutrition needs of mammalian cells in tissue culture. Science. 1955;122:501–4. 10.1126/science.122.3168.501. 13255879 10.1126/science.122.3168.501

[9] Bannai S, Tsukeda H, Okumura H. Effect of antioxidants on cultured human diploid fibroblasts exposed to cystine-free medium. Biochem Biophys Res Commun. 1977;74:1582–8. 10.1016/0006-291X(77)90623-4. 843380 10.1016/0006-291x(77)90623-4

[10] Bannai S, Kitamura E. Transport interaction of L-cystine and L-glutamate in human diploid fibroblasts in culture. J Biol Chem. 1980;255:2372–6. 7358676

[11] Ursini F, Maiorino M. Lipid peroxidation and ferroptosis: the role of GSH and GPx4. Free Radic Biol Med. 2020;152:175–85. 10.1016/j.freeradbiomed.2020.02.027. 32165281 10.1016/j.freeradbiomed.2020.02.027

[12] Ursini F, Maiorino M, Valente M, Ferri L, Gregolin C. Purification from pig liver of a protein which protects liposomes and biomembranes from peroxidative degradation and exhibits glutathione peroxidase activity on phosphatidylcholine hydroperoxides. Biochim Biophys Acta (BBA) Lipids Lipid Metab. 1982;710:197–211. 10.1016/0005-2760(82)90150-3. 10.1016/0005-2760(82)90150-3 7066358

[13] Moi P, Chan K, Asunis I, Cao A, Kan YW. Isolation of NF-E2-related factor 2 (Nrf2), a NF-E2-like basic leucine zipper transcriptional activator that binds to the tandem NF-E2/AP1 repeat of the beta-globin locus control region. Proc Natl Acad Sci. 1994;91:9926–30. 10.1073/pnas.91.21.9926. 7937919 10.1073/pnas.91.21.9926 PMC44930

[14] Itoh K, Chiba T, Takahashi S, Ishii T, Igarashi K, Katoh Y, et al. An Nrf2/small maf heterodimer mediates the induction of phase ii detoxifying enzyme genes through antioxidant response elements. Biochem Biophys Res Commun. 1997;236:313–22. 10.1006/bbrc.1997.6943. 9240432 10.1006/bbrc.1997.6943

[15] Habib E, Linher-Melville K, Lin HX, Singh G. Expression of xCT and activity of system xc− are regulated by NRF2 in human breast cancer cells in response to oxidative stress. Redox Biol. 2015;5:33–42. 10.1016/j.redox.2015.03.003. 25827424 10.1016/j.redox.2015.03.003 PMC4392061

[16] Sasaki H, Sato H, Kuriyama-Matsumura K, Sato K, Maebara K, Wang H, et al. Electrophile response element-mediated induction of the cystine/glutamate exchange transporter gene expression. J Biol Chem. 2002;277:44765–71. 10.1074/jbc.m208704200. 12235164 10.1074/jbc.M208704200

[17] Dodson M, Castro-Portuguez R, Zhang DD. NRF2 plays a critical role in mitigating lipid peroxidation and ferroptosis. Redox Biol. 2019;23:101107. 10.1016/j.redox.2019.101107. 30692038 10.1016/j.redox.2019.101107 PMC6859567

[18] Alam J, Stewart D, Touchard C, Boinapally S, Choi AMK, Cook JL. Nrf2, a Cap’n’Collar transcription factor, regulates induction of the heme oxygenase-1 gene. J Biol Chem. 1999;274:26071–8. 10.1074/jbc.274.37.26071. 10473555 10.1074/jbc.274.37.26071

[19] Chorley BN, Campbell MR, Wang X, Karaca M, Sambandan D, Bangura F, et al. Identification of novel NRF2-regulated genes by ChIP-Seq: influence on retinoid X receptor alpha. Nucleic Acids Res. 2012;40:7416–29. 10.1093/nar/gks409. 22581777 10.1093/nar/gks409 PMC3424561

[20] Sakurai A, Nishimoto M, Himeno S, Imura N, Tsujimoto M, Kunimoto M, et al. Transcriptional regulation of thioredoxin reductase 1 expression by cadmium in vascular endothelial cells: role of NF-E2-related factor-2. J Cell Physiol. 2005;203:529–37. 10.1002/jcp.20246. 15521073 10.1002/jcp.20246

[21] Rocha CRR, Garcia CCM, Vieira DB, Quinet A, De Andrade-Lima LC, Munford V, et al. Glutathione depletion sensitizes cisplatin- and temozolomide-resistant glioma cells in vitro and in vivo. Cell Death Dis. 2014;5:e1505–e1505. 10.1038/cddis.2014.465. 25356874 10.1038/cddis.2014.465 PMC4649538

[22] Rojo de la Vega M, Chapman E, Zhang DD. NRF2 and the hallmarks of cancer. Cancer Cell. 2018;34:21–43. 10.1016/j.ccell.2018.03.022. 29731393 10.1016/j.ccell.2018.03.022 PMC6039250

[23] Dolma S, Lessnick SL, Hahn WC, Stockwell BR. Identification of genotype-selective antitumor agents using synthetic lethal chemical screening in engineered human tumor cells. Cancer Cell. 2003;3:285–96. 10.1016/S1535-6108(03)00050-3. 12676586 10.1016/s1535-6108(03)00050-3

[24] Yagoda N, von Rechenberg M, Zaganjor E, Bauer AJ, Yang WS, Fridman DJ, et al. RAS–RAF–MEK-dependent oxidative cell death involving voltage-dependent anion channels. Nature. 2007;447:865–9. 10.1038/nature05859. 10.1038/nature05859 PMC3047570 17568748

[25] Yang WS, Stockwell BR. Synthetic lethal screening identifies compounds activating iron-dependent, nonapoptotic cell death in oncogenic-RAS-harboring cancer cells. Chem Biol. 2008;15:234–45. 10.1016/j.chembiol.2008.02.010. 18355723 10.1016/j.chembiol.2008.02.010 PMC2683762

[26] Seiler A, Schneider M, Förster H, Roth S, Wirth EK, Culmsee C, et al. Glutathione peroxidase 4 senses and translates oxidative stress into 12/15-lipoxygenase dependent- and AIF-mediated cell death. Cell Metab. 2008;8:237–48. 10.1016/j.cmet.2008.07.005. 18762024 10.1016/j.cmet.2008.07.005

[27] Banjac A, Perisic T, Sato H, Seiler A, Bannai S, Weiss N, et al. The cystine/cysteine cycle: a redox cycle regulating susceptibility versus resistance to cell death. Oncogene. 2008;27:1618–28. 10.1038/sj.onc.1210796. 17828297 10.1038/sj.onc.1210796

[28] Dixon JS, Lemberg MK, Lamprecht RM, Skouta R, Zaitsev ME, Gleason EC, et al. Ferroptosis: an iron-dependent form of nonapoptotic cell death. Cell. 2012;149:1060–72. 10.1016/j.cell.2012.03.042. 22632970 10.1016/j.cell.2012.03.042 PMC3367386

[29] Dixon SJ, Patel DN, Welsch M, Skouta R, Lee ED, Hayano M, et al. Pharmacological inhibition of cystine–glutamate exchange induces endoplasmic reticulum stress and ferroptosis. Elife. 2014; 3. 10.7554/elife.02523. 10.7554/eLife.02523 PMC4054777 24844246

[30] Kaspar JW, Niture SK, Jaiswal AK. Nrf2:INrf2 (Keap1) signaling in oxidative stress. Free Radic Biol Med. 2009;47:1304–9. 10.1016/j.freeradbiomed.2009.07.035. 19666107 10.1016/j.freeradbiomed.2009.07.035 PMC2763938

[31] Tonelli C, Chio IIC, Tuveson DA. Transcriptional regulation by Nrf2. Antioxid Redox Signal. 2018;29:1727–45. 10.1089/ars.2017.7342. 28899199 10.1089/ars.2017.7342 PMC6208165

[32] Komatsu M, Kurokawa H, Waguri S, Taguchi K, Kobayashi A, Ichimura Y, et al. The selective autophagy substrate p62 activates the stress responsive transcription factor Nrf2 through inactivation of Keap1. Nat Cell Biol. 2010;12:213–23. 10.1038/ncb2021. 20173742 10.1038/ncb2021

[33] Jaramillo MC, Zhang DD. The emerging role of the Nrf2–Keap1 signaling pathway in cancer. Genes Dev. 2013;27:2179–91. 10.1101/gad.225680.113. 24142871 10.1101/gad.225680.113 PMC3814639

[34] Friedmann Angeli JP, Schneider M, Proneth B, Tyurina YY, Tyurin VA, Hammond VJ, et al. Inactivation of the ferroptosis regulator Gpx4 triggers acute renal failure in mice. Nat Cell Biol. 2014;16:1180–91. 10.1038/ncb3064. 25402683 10.1038/ncb3064 PMC4894846

[35] dos Santos AF, Fazeli G, Xavier da Silva TN, Friedmann Angeli JP. Ferroptosis: mechanisms and implications for cancer development and therapy response. Trends Cell Biol. 2023;33:1062–76. 10.1016/j.tcb.2023.04.005. 37230924 10.1016/j.tcb.2023.04.005

[36] Lee J, Roh JL. Targeting iron-sulfur clusters in cancer: opportunities and challenges for ferroptosis-based therapy. Cancers. 2023;15:2694 10.3390/cancers15102694. 37345031 10.3390/cancers15102694 PMC10216707

[37] Yang SW, Sriramaratnam R, Welsch EM, Shimada K, Skouta R, Viswanathan SV, et al. Regulation of ferroptotic cancer cell death by GPX4. Cell. 2014;156:317–31. 10.1016/j.cell.2013.12.010. 24439385 10.1016/j.cell.2013.12.010 PMC4076414

[38] Dixon SJ, Olzmann JA. The cell biology of ferroptosis. Nat Rev Mol Cell Biol. 2024. 10.1038/s41580-024-00703-5. 10.1038/s41580-024-00703-5 PMC12187608 38366038

[39] Dixon SJ, Stockwell BR. The role of iron and reactive oxygen species in cell death. Nat Chem Biol. 2014;10:9–17. 10.1038/nchembio.1416. 24346035 10.1038/nchembio.1416

[40] Dixon SJ, Winter GE, Musavi LS, Lee ED, Snijder B, Rebsamen M, et al. Human haploid cell genetics reveals roles for lipid metabolism genes in nonapoptotic cell death. ACS Chem Biol. 2015;10:1604–9. 10.1021/acschembio.5b00245. 25965523 10.1021/acschembio.5b00245 PMC4509420

[41] Fan Z, Wirth AK, Chen D, Wruck CJ, Rauh M, Buchfelder M, et al. Nrf2-Keap1 pathway promotes cell proliferation and diminishes ferroptosis. Oncogenesis. 2017;6:e371–e371. 10.1038/oncsis.2017.65. 28805788 10.1038/oncsis.2017.65 PMC5608917

[42] Emmanuel N, Li H, Chen J, Zhang Y. FSP1, a novel KEAP1/NRF2 target gene regulating ferroptosis and radioresistance in lung cancers. Oncotarget. 2022;13:1136–9. 10.18632/oncotarget.28301. 36264074 10.18632/oncotarget.28301 PMC9584440

[43] Dibra D, Xiong S, Moyer SM, El-Naggar AK, Qi Y, Su X, et al. Mutant p53 protects triple-negative breast adenocarcinomas from ferroptosis in vivo. Sci Adv. 2024;10. 10.1126/sciadv.adk1835. 10.1126/sciadv.adk1835 PMC10866549 38354236

[44] Yang Z, Zou S, Zhang Y, Zhang J, Zhang P, Xiao L, et al. ACTL6A protects gastric cancer cells against ferroptosis through induction of glutathione synthesis. Nat Commun. 2023;14:4193. 10.1038/s41467-023-39901-8. 37443154 10.1038/s41467-023-39901-8 PMC10345109

[45] He X, Zhou Y, Chen W, Zhao X, Duan L, Zhou H, et al. Repurposed pizotifen malate targeting NRF2 exhibits anti-tumor activity through inducing ferroptosis in esophageal squamous cell carcinoma. Oncogene. 2023;42:1209–23. 10.1038/s41388-023-02636-3. 36841865 10.1038/s41388-023-02636-3

[46] Mao C, Liu X, Zhang Y, Lei G, Yan Y, Lee H, et al. DHODH-mediated ferroptosis defence is a targetable vulnerability in cancer. Nature. 2021;593:586–90. 10.1038/s41586-021-03539-7. 33981038 10.1038/s41586-021-03539-7 PMC8895686

[47] Koppula P, Lei G, Zhang Y, Yan Y, Mao C, Kondiparthi L, et al. A targetable CoQ-FSP1 axis drives ferroptosis- and radiation-resistance in KEAP1 inactive lung cancers. Nat Commun. 2022;13:2206. 10.1038/s41467-022-29905-1. 35459868 10.1038/s41467-022-29905-1 PMC9033817

[48] Doll S, Freitas FP, Shah R, Aldrovandi M, da Silva MC, Ingold I, et al. FSP1 is a glutathione-independent ferroptosis suppressor. Nature. 2019;575:693–8. 10.1038/s41586-019-1707-0. 31634899 10.1038/s41586-019-1707-0

[49] Kraft VAN, Bezjian CT, Pfeiffer S, Ringelstetter L, Müller C, Zandkarimi F, et al. GTP cyclohydrolase 1/tetrahydrobiopterin counteract ferroptosis through lipid remodeling. ACS Cent Sci. 2020;6:41–53. 10.1021/acscentsci.9b01063. 31989025 10.1021/acscentsci.9b01063 PMC6978838

[50] Soula M, Weber RA, Zilka O, Alwaseem H, La K, Yen F, et al. Metabolic determinants of cancer cell sensitivity to canonical ferroptosis inducers. Nat Chem Biol. 2020;16:1351–60. 10.1038/s41589-020-0613-y. 32778843 10.1038/s41589-020-0613-y PMC8299533

[51] Shimada K, Skouta R, Kaplan A, Yang WS, Hayano M, Dixon SJ, et al. Global survey of cell death mechanisms reveals metabolic regulation of ferroptosis. Nat Chem Biol. 2016;12:497–503. 10.1038/nchembio.2079. 27159577 10.1038/nchembio.2079 PMC4920070

[52] Yin H, Xu L, Porter NA. Free radical lipid peroxidation: mechanisms and analysis. Chem Rev. 2011;111:5944–72. 10.1021/cr200084z. 21861450 10.1021/cr200084z

[53] Pope LE, Dixon SJ. Regulation of ferroptosis by lipid metabolism. Trends Cell Biol. 2023;33:1077–87. 10.1016/j.tcb.2023.05.003. 37407304 10.1016/j.tcb.2023.05.003 PMC10733748

[54] Doll S, Proneth B, Tyurina YY, Panzilius E, Kobayashi S, Ingold I, et al. ACSL4 dictates ferroptosis sensitivity by shaping cellular lipid composition. Nat Chem Biol. 2017;13:91–8. 10.1038/nchembio.2239. 27842070 10.1038/nchembio.2239 PMC5610546

[55] Liang D, Feng Y, Zandkarimi F, Wang H, Zhang Z, Kim J, et al. Ferroptosis surveillance independent of GPX4 and differentially regulated by sex hormones. Cell. 2023;186:2748–2764.e22. 10.1016/j.cell.2023.05.003. 37267948 10.1016/j.cell.2023.05.003 PMC10330611

[56] Freitas FP, Alborzinia H, dos Santos AF, Nepachalovich P, Pedrera L, Zilka O, et al. 7-Dehydrocholesterol is an endogenous suppressor of ferroptosis. Nature. 2024;626:401–10. 10.1038/s41586-023-06878-9. 38297129 10.1038/s41586-023-06878-9

[57] Li Y, Ran Q, Duan Q, Jin J, Wang Y, Yu L, et al. 7-Dehydrocholesterol dictates ferroptosis sensitivity. Nature. 2024;626:411–8. 10.1038/s41586-023-06983-9. 38297130 10.1038/s41586-023-06983-9 PMC11298758

[58] Yang WS, Kim KJ, Gaschler MM, Patel M, Shchepinov MS, Stockwell BR. Peroxidation of polyunsaturated fatty acids by lipoxygenases drives ferroptosis. Proc Natl Acad Sci. 2016;113. 10.1073/pnas.1603244113. 10.1073/pnas.1603244113 PMC5003261 27506793

[59] Stockwell BR. Ferroptosis turns 10: Emerging mechanisms, physiological functions, and therapeutic applications. Cell. 2022;185:2401–21. 10.1016/j.cell.2022.06.003. 35803244 10.1016/j.cell.2022.06.003 PMC9273022

[60] Kuang F, Liu J, Xie Y, Tang D, Kang R. MGST1 is a redox-sensitive repressor of ferroptosis in pancreatic cancer cells. Cell Chem Biol. 2021;28:765–775.e5. 10.1016/j.chembiol.2021.01.006. 33539732 10.1016/j.chembiol.2021.01.006

[61] Sen U, Coleman C, Gandhi N, Jethalia V, Demircioglu D, Elliott A, et al. SCD1 inhibition blocks the AKT–NRF2–SLC7A11 pathway to induce lipid metabolism remodeling and ferroptosis priming in lung adenocarcinoma. Cancer Res. 2025;85:2485–503. 10.1158/0008-5472.CAN-24-2745. 40198901 10.1158/0008-5472.CAN-24-2745 PMC12221774

[62] Shi W, Ong YQ, Majee P, Wong ES, Velazquez AMV, Fidan K, et al. Pirin transcriptionally regulates PLA2G4A to inhibit ferroptosis in colorectal cancer via lipid profile remodeling. Adv Sci. 2025. 10.1002/advs.202516385. 10.1002/advs.202516385 PMC12931218 41400081

[63] Hassannia B, Vandenabeele P, Vanden Berghe T. Targeting ferroptosis to iron out cancer. Cancer Cell. 2019;35:830–49. 10.1016/j.ccell.2019.04.002. 31105042 10.1016/j.ccell.2019.04.002

[64] Kakhlon O, Cabantchik ZI. The labile iron pool: characterization, measurement, and participation in cellular processes(1). Free Radic Biol Med. 2002;33:1037–46. 10.1016/S0891-5849(02)01006-7. 12374615 10.1016/s0891-5849(02)01006-7

[65] Galy B, Conrad M, Muckenthaler M. Mechanisms controlling cellular and systemic iron homeostasis. Nat Rev Mol Cell Biol. 2024;25:133–55. 10.1038/s41580-023-00648-1. 37783783 10.1038/s41580-023-00648-1

[66] Mancias JD, Pontano Vaites L, Nissim S, Biancur DE, Kim AJ, Wang X, et al. Ferritinophagy via NCOA4 is required for erythropoiesis and is regulated by iron dependent HERC2-mediated proteolysis. Elife. 2015;4. 10.7554/eLife.10308. 10.7554/eLife.10308 PMC4592949 26436293

[67] Wang J, Wu N, Peng M, Oyang L, Jiang X, Peng Q, et al. Ferritinophagy: research advance and clinical significance in cancers. Cell Death Discov. 2023;9:463 10.1038/s41420-023-01753-y. 38110359 10.1038/s41420-023-01753-y PMC10728094

[68] Hassannia B, Wiernicki B, Ingold I, Qu F, Van Herck S, Tyurina YY, et al. Nano-targeted induction of dual ferroptotic mechanisms eradicates high-risk neuroblastoma. J Clin Investig. 2018;128:3341–55. 10.1172/jci99032. 29939160 10.1172/JCI99032 PMC6063467

[69] Anandhan A, Dodson M, Schmidlin CJ, Liu P, Zhang DD. Breakdown of an ironclad defense system: the critical role of NRF2 in mediating ferroptosis. Cell Chem Biol. 2020;27:436–47. 10.1016/j.chembiol.2020.03.011. 32275864 10.1016/j.chembiol.2020.03.011 PMC7597851

[70] Kerins MJ, Ooi A. The roles of NRF2 in modulating cellular iron homeostasis. Antioxid Redox Signal. 2018;29:1756–73. 10.1089/ars.2017.7176. 28793787 10.1089/ars.2017.7176 PMC6208163

[71] Yu X, Wang Y, Tan J, Li Y, Yang P, Liu X, et al. Inhibition of NRF2 enhances the acute myeloid leukemia cell death induced by venetoclax via the ferroptosis pathway. Cell Death Discov. 2024;10:35 10.1038/s41420-024-01800-2. 38238299 10.1038/s41420-024-01800-2 PMC10796764

[72] Namgaladze D, Fuhrmann DC, Brüne B. Interplay of Nrf2 and BACH1 in inducing ferroportin expression and enhancing resistance of human macrophages towards ferroptosis. Cell Death Discov. 2022;8:327 10.1038/s41420-022-01117-y. 35853860 10.1038/s41420-022-01117-y PMC9296510

[73] Anandhan A, Dodson M, Shakya A, Chen J, Liu P, Wei Y, et al. NRF2 controls iron homeostasis and ferroptosis through HERC2 and VAMP8. Sci Adv. 2023;9. 10.1126/sciadv.ade9585. 10.1126/sciadv.ade9585 PMC9891695 36724221

[74] Li Y, Xu B, Ren X, Wang L, Xu Y, Zhao Y, et al. Inhibition of CISD2 promotes ferroptosis through ferritinophagy-mediated ferritin turnover and regulation of p62–Keap1–NRF2 pathway. Cell Mol Biol Lett. 2022;27:81 10.1186/s11658-022-00383-z. 36180832 10.1186/s11658-022-00383-z PMC9523958

[75] Cao JY, Poddar A, Magtanong L, Lumb JH, Mileur TR, Reid MA, et al. A genome-wide haploid genetic screen identifies regulators of glutathione abundance and ferroptosis sensitivity. Cell Rep. 2019;26:1544–1556.e8. 10.1016/j.celrep.2019.01.043. 30726737 10.1016/j.celrep.2019.01.043 PMC6424331

[76] Lin H, Chen X, Zhang C, Yang T, Deng Z, Song Y, et al. EF24 induces ferroptosis in osteosarcoma cells through HMOX1. Biomed. Pharmacother. 2021;136:111202. 10.1016/j.biopha.2020.111202. 33453607 10.1016/j.biopha.2020.111202

[77] Ren T, Huang J, Sun W, Wang G, Wu Y, Jiang Z, et al. Zoledronic acid induces ferroptosis by reducing ubiquinone and promoting HMOX1 expression in osteosarcoma cells. Front Pharmacol. 2023;13. 10.3389/fphar.2022.1071946. 10.3389/fphar.2022.1071946 PMC9846057 36686696

[78] Chang LC, Chiang SK, Chen SE, Yu YL, Chou RH, Chang WC. Heme oxygenase-1 mediates BAY 11–7085 induced ferroptosis. Cancer Lett. 2018;416:124–37. 10.1016/j.canlet.2017.12.025. 29274359 10.1016/j.canlet.2017.12.025

[79] Zheng C, Zhang B, Li Y, Liu K, Wei W, Liang S, et al. Donafenib and GSK-J4 synergistically induce ferroptosis in liver cancer by upregulating HMOX1 expression. Adv. Sci. 2023;10. 10.1002/advs.202206798. 10.1002/advs.202206798 PMC10401117 37330650

[80] Yang C, Wang T, Zhao Y, Meng X, Ding W, Wang Q, et al. Flavonoid 4,4′-dimethoxychalcone induced ferroptosis in cancer cells by synergistically activating Keap1/Nrf2/HMOX1 pathway and inhibiting FECH. Free Radic Biol Med. 2022;188:14–23. 10.1016/j.freeradbiomed.2022.06.010. 35697292 10.1016/j.freeradbiomed.2022.06.010

[81] Wang Z, Ma J, Li X, Wu Y, Shi H, Chen Y, et al. Quercetin induces p53-independent cancer cell death through lysosome activation by the transcription factor EB and reactive oxygen species-dependent ferroptosis. Br J Pharm. 2021;178:1133–48. 10.1111/bph.15350. 10.1111/bph.15350 33347603

[82] Wang Z, Li W, Wang X, Zhu Q, Liu L, Qiu S, et al. Isoliquiritigenin induces HMOX1 and GPX4-mediated ferroptosis in gallbladder cancer cells. Chin Med J. 2023;136:2210–20. 10.1097/CM9.0000000000002675. 37488674 10.1097/CM9.0000000000002675 PMC10508381

[83] Zhang C, Liu X, Jin S, Chen Y, Guo R Ferroptosis in cancer therapy: a novel approach to reversing drug resistance. Mol Cancer. 2022;21. 10.1186/s12943-022-01530-y. 10.1186/s12943-022-01530-y PMC8840702 35151318

[84] Reichard JF, Sartor MA, Puga A. BACH1 is a specific repressor of HMOX1 that is inactivated by arsenite. J Biol Chem. 2008;283:22363–70. 10.1074/jbc.M801784200. 18550526 10.1074/jbc.M801784200 PMC2504887

[85] Cole SPC. Multidrug resistance protein 1 (MRP1, ABCC1), a “Multitasking” ATP-binding cassette (ABC) transporter. J Biol Chem. 2014;289:30880–8. 10.1074/jbc.R114.609248. 25281745 10.1074/jbc.R114.609248 PMC4223294

[86] Ji L, Li H, Gao P, Shang G, Zhang DD, Zhang N, et al. Nrf2 pathway regulates multidrug-resistance-associated protein 1 in small cell lung cancer. PLoS ONE. 2013;8:e63404 10.1371/journal.pone.0063404. 23667609 10.1371/journal.pone.0063404 PMC3646742

[87] Hanssen KM, Haber M, Fletcher JI. Targeting multidrug resistance-associated protein 1 (MRP1)-expressing cancers: beyond pharmacological inhibition. Drug Resist Updates. 2021;59:100795. 10.1016/j.drup.2021.100795. 10.1016/j.drup.2021.100795 34983733

[88] Ichihara G, Katsumata Y, Sugiura Y, Matsuoka Y, Maeda R, Endo J, et al. MRP1-dependent extracellular release of glutathione induces cardiomyocyte ferroptosis after ischemia-reperfusion. Circ Res. 2023;133:861–76. 10.1161/CIRCRESAHA.123.323517. 37818671 10.1161/CIRCRESAHA.123.323517

[89] Shi Y, Xu N, Liu B, Ma Y, Fu X, Shang Y, et al. Mifepristone protects acetaminophen induced liver injury through NRF2/GSH/GST mediated ferroptosis suppression. Free Radic Biol Med. 2024;222:229–43. 10.1016/j.freeradbiomed.2024.06.014. 38906233 10.1016/j.freeradbiomed.2024.06.014

[90] de Souza I, Monteiro LKS, Guedes CB, Silva MM, Andrade-Tomaz M, Contieri B, et al. High levels of NRF2 sensitize temozolomide-resistant glioblastoma cells to ferroptosis via ABCC1/MRP1 upregulation. Cell Death Dis. 2022;13:591. 10.1038/s41419-022-05044-9. 35803910 10.1038/s41419-022-05044-9 PMC9270336

[91] Liu S. Aryl hydrocarbon receptor alleviates hepatic fibrosis by inducing hepatic stellate cell ferroptosis. J Cell Mol Med. 2024;28. 10.1111/jcmm.70278. 10.1111/jcmm.70278 PMC11628353 39654034

[92] Zhao J, An H, Zhou S, Tian T, Yao Y, Shi L. Multidrug resistance-associated protein 1 aberration-incurred glutathione efflux drives renal ferroptosis and acute kidney injury-chronic kidney disease progression. J Cell Physiol. 2025;240. 10.1002/jcp.70105. 10.1002/jcp.70105 41139230

[93] Liang Y, Ye F, Xu C, Zou L, Hu Y, Hu J, et al. A novel survival model based on a Ferroptosis-related gene signature for predicting overall survival in bladder cancer. BMC Cancer. 2021;21:943. 10.1186/s12885-021-08687-7. 34418989 10.1186/s12885-021-08687-7 PMC8380338

[94] Jin Y, Wang Z, He D, Zhu Y, Gong L, Xiao M, et al. Analysis of ferroptosis-mediated modification patterns and tumor immune microenvironment characterization in uveal melanoma. Front Cell Dev Biol. 2021;9. 10.3389/fcell.2021.685120. 10.3389/fcell.2021.685120 PMC8353259 34386492

[95] Chen S, Diao J, Yue Z, Wei R. Identification and validation of ferroptosis-related genes and immune cell infiltration in thyroid associated ophthalmopathy. Front Genet. 2023;14. 10.3389/fgene.2023.1118391. 10.3389/fgene.2023.1118391 PMC10067720 37021001

[96] Viswanathan VS, Ryan MJ, Dhruv HD, Gill S, Eichhoff OM, Seashore-Ludlow B, et al. Dependency of a therapy-resistant state of cancer cells on a lipid peroxidase pathway. Nature. 2017;547:453–7. 10.1038/nature23007. 28678785 10.1038/nature23007 PMC5667900

[97] Yan HF, Zou T, Tuo QZ, Xu S, Li H, Belaidi AA, et al. Ferroptosis: mechanisms and links with diseases. Signal Transduct Target Ther. 2021;6(1):49. 10.1038/s41392-020-00428-9. 33536413 10.1038/s41392-020-00428-9 PMC7858612

[98] Buccarelli M, Marconi M, Pacioni S, De Pasqualis I, D’Alessandris QG, Martini M, et al. Inhibition of autophagy increases susceptibility of glioblastoma stem cells to temozolomide by igniting ferroptosis. Cell Death Dis. 2018;9.10.1038/s41419-018-0864-7. 10.1038/s41419-018-0864-7 PMC6079099 30082680

[99] Wang Y, Yan S, Liu X, Deng F, Wang P, Yang L, et al. PRMT4 promotes ferroptosis to aggravate doxorubicin-induced cardiomyopathy via inhibition of the Nrf2/GPX4 pathway. Cell Death Differ. 2022;29:1982–95. 10.1038/s41418-022-00990-5. 35383293 10.1038/s41418-022-00990-5 PMC9525272

[100] Zhang Y, Tan H, Daniels JD, Zandkarimi F, Liu H, Brown LM, et al. Imidazole Ketone Erastin Induces Ferroptosis and Slows Tumor Growth in a Mouse Lymphoma Model. Cell Chem Biol. 2019;26:623–633.e9. 10.1016/j.chembiol.2019.01.008. 30799221 10.1016/j.chembiol.2019.01.008 PMC6525071

[101] Liu H, Forouhar F, Lin AJ, Wang Q, Polychronidou V, Soni RK, et al. Small-molecule allosteric inhibitors of GPX4. Cell Chem Biol. 2022;29(12):1680–1693.e9. 10.1016/j.chembiol.2022.11.003. 36423641 10.1016/j.chembiol.2022.11.003 PMC9772252

[102] Yang B, Pan J, Zhang XN, Wang H, He L, Rong X, et al. NRF2 activation suppresses motor neuron ferroptosis induced by the SOD1G93A mutation and exerts neuroprotection in amyotrophic lateral sclerosis. Neurobiol Dis. 2023;184:106210. 10.1016/j.nbd.2023.106210. 37352984 10.1016/j.nbd.2023.106210

[103] Phillips E, Penate-Medina O, Zanzonico PB, Carvajal RD, Mohan P, Ye Y, et al. Clinical translation of an ultrasmall inorganic optical-PET imaging nanoparticle probe. Sci Transl Med. 2014;6. 10.1126/scitranslmed.3009524. 10.1126/scitranslmed.3009524 PMC4426391 25355699

[104] Ma P, Xiao H, Yu C, Liu J, Cheng Z, Song H, et al. Enhanced cisplatin chemotherapy by iron oxide nanocarrier-mediated generation of highly toxic reactive oxygen species. Nano Lett. 2017;17:928–37. 10.1021/acs.nanolett.6b04269. 28139118 10.1021/acs.nanolett.6b04269

[105] Song R, Li T, Ye J, Sun F, Hou B, Saeed M, et al. Acidity-activatable dynamic nanoparticles boosting ferroptotic cell death for immunotherapy of cancer. Adv Mater. 2021;33. 10.1002/adma.202101155. 10.1002/adma.202101155 34170581

[106] Nie Q, Hu Y, Yu X, Li X, Fang X. Induction and application of ferroptosis in cancer therapy. Cancer Cell Int. 2022;22:12. 10.1186/s12935-021-02366-0. 10.1186/s12935-021-02366-0 PMC8742449 34996454

[107] Ren D, Villeneuve NF, Jiang T, Wu T, Lau A, Toppin HA, et al. Brusatol enhances the efficacy of chemotherapy by inhibiting the Nrf2-mediated defense mechanism. Proc Natl Acad Sci. 2011;108:1433–8. 10.1073/pnas.1014275108. 21205897 10.1073/pnas.1014275108 PMC3029730

[108] Roy N, Wyseure T, Lo IC, Lu J, Eissler CL, Bernard SM, et al. A covalent allosteric molecular glue suppresses NRF2-dependent cancer growth. Cancer Discov. 2025. 10.1158/2159-8290.CD-25-1187. 10.1158/2159-8290.CD-25-1187 PMC13133613 41417010

[109] Zhuang C, Wu Z, Xing C, Miao Z. Small molecules inhibiting Keap1–Nrf2 protein–protein interactions: a novel approach to activate Nrf2 function. Medchemcomm. 2017;8:286–94. 10.1039/C6MD00500D. 30108745 10.1039/c6md00500d PMC6072482

[110] Ishida T, Ciulli A. E3 ligase ligands for PROTACs: how they were found and how to discover new ones. SLAS Discov. 2021;26:484–502. 10.1177/2472555220965528. 33143537 10.1177/2472555220965528 PMC8013866

[111] Dinkova-Kostova AT, Copple IM. Advances and challenges in therapeutic targeting of NRF2. Trends Pharm Sci. 2023;44:137–49. 10.1016/j.tips.2022.12.003. 36628798 10.1016/j.tips.2022.12.003

[112] Baird L, Zhang L, Hidaka T, Xi L, Wang K, Tateno K, et al. Systemic activation of NRF2 contributes to the therapeutic efficacy of clinically-approved KRAS-G12C anti-cancer drugs. Br J Cancer. 2025;133:1377–90. 10.1038/s41416-025-03162-7. 40890297 10.1038/s41416-025-03162-7 PMC12572401

[113] Yang B, Pan J, Zhang XN, Wang H, He L, Rong X, et al. NRF2 activation suppresses motor neuron ferroptosis induced by the SOD1G93A mutation and exerts neuroprotection in amyotrophic lateral sclerosis. Neurobiol Dis. 2023;184:106210. 10.1016/j.nbd.2023.106210. 37352984 10.1016/j.nbd.2023.106210

[114] Doss JF, Jonassaint JC, Garrett ME, Ashley-Koch AE, Telen MJ, Chi JT. Phase 1 study of a sulforaphane-containing broccoli sprout homogenate for sickle cell disease. PLoS ONE. 2016;11:e0152895 10.1371/journal.pone.0152895. 27071063 10.1371/journal.pone.0152895 PMC4829228

[115] Sugiyama A, Ohta T, Obata M, Takahashi K, Seino M, Nagase S. xCT inhibitor sulfasalazine depletes paclitaxel-resistant tumor cells through ferroptosis in uterine serous carcinoma. Oncol Lett. 2020;20:2689–700. 10.3892/ol.2020.11813. 32782585 10.3892/ol.2020.11813 PMC7400102

[116] Zheng J, Sato M, Mishima E, Sato H, Proneth B, Conrad M. Sorafenib fails to trigger ferroptosis across a wide range of cancer cell lines. Cell Death Dis. 2021;12:698. 10.1038/s41419-021-03998-w. 34257282 10.1038/s41419-021-03998-w PMC8277867

[117] Keating GM. Sorafenib: a review in hepatocellular carcinoma. Target Oncol. 2017;12:243–53. 10.1007/s11523-017-0484-7. 28299600 10.1007/s11523-017-0484-7

[118] Coriat R, Nicco C, Chéreau C, Mir O, Alexandre J, Ropert S, et al. Sorafenib-induced hepatocellular carcinoma cell death depends on reactive oxygen species production in vitro and in vivo. Mol Cancer Ther. 2012;11:2284–93. 10.1158/1535-7163.mct-12-0093. 22902857 10.1158/1535-7163.MCT-12-0093

[119] Zhang LH, Tang M, Tao X, Shao Q, Thomas V, Shimizu S, et al. Covalent targeting of glutamate cysteine ligase to inhibit glutathione synthesis**. ChemBioChem. 2023;24. 10.1002/cbic.202300371. 10.1002/cbic.202300371 PMC10739677 37756477

[120] Sun Y, Zheng Y, Wang C, Liu Y. Glutathione depletion induces ferroptosis, autophagy, and premature cell senescence in retinal pigment epithelial cells. Cell Death Dis. 2018;9. 10.1038/s41419-018-0794-4. 10.1038/s41419-018-0794-4 PMC6037763 29988039

[121] Chen Z, Niu K, Li M, Deng Y, Zhang J, Wei D, et al. GCLC desuccinylation regulated by oxidative stress protects human cancer cells from ferroptosis. Cell Death Differ. 2025;32:1679–90. 10.1038/s41418-025-01505-8. 40188196 10.1038/s41418-025-01505-8 PMC12432198

[122] Yang Z, Han T, Yang R, Zhang Y, Qin Y, Hou J, et al. Dicoumarol sensitizes hepatocellular carcinoma cells to ferroptosis induced by imidazole ketone erastin. Front Immunol. 2025;16. 10.3389/fimmu.2025.1531874. 10.3389/fimmu.2025.1531874 PMC11852437 40007539

[123] Yuan Z, Wang X, Qin B, Hu R, Miao R, Zhou Y, et al. Targeting NQO1 induces ferroptosis and triggers anti-tumor immunity in immunotherapy-resistant KEAP1-deficient cancers. Drug Resist Updates. 2024;77:101160. 10.1016/j.drup.2024.101160. 10.1016/j.drup.2024.101160 39490240

[124] Tang C, He C, Wang D, Guo J, Yin X, Ye H, et al. Co-delivery of sorafenib and an FSP1 inhibitor triggers dual ferroptosis in tumor cells and immunosuppressive macrophages for enhanced immunotherapy in mouse models of hepatocellular carcinoma. Nat Commun. 2025;16:10096. 10.1038/s41467-025-65056-9. 41253833 10.1038/s41467-025-65056-9 PMC12627561

[125] Nakamura T, Mishima E, Yamada N, Mourão ASD, Trümbach D, Doll S, et al. Integrated chemical and genetic screens unveil FSP1 mechanisms of ferroptosis regulation. Nat Struct Mol Biol. 2023;30:1806–15. 10.1038/s41594-023-01136-y. 37957306 10.1038/s41594-023-01136-y PMC10643123

[126] Xavier da Silva TN, Schulte C, Alves AN, Maric HM, Friedmann Angeli JP. Molecular characterization of AIFM2/FSP1 inhibition by iFSP1-like molecules. Cell Death Dis. 2023;14:281. 10.1038/s41419-023-05787-z. 37080964 10.1038/s41419-023-05787-z PMC10119282

[127] Mai TT, Hamaï A, Hienzsch A, Cañeque T, Müller S, Wicinski J, et al. Salinomycin kills cancer stem cells by sequestering iron in lysosomes. Nat Chem. 2017;9:1025–33. 10.1038/nchem.2778. 28937680 10.1038/nchem.2778 PMC5890907

[128] Naujokat C, Steinhart R. Salinomycin as a drug for targeting human cancer stem cells. J Biomed Biotechnol. 2012;2012:1–17. 10.1155/2012/950658. 23251084 10.1155/2012/950658 PMC3516046

[129] Thomas CM, Stauffer WM, Alpern JD. Food and drug administration approval of artesunate for severe malaria: Enough to achieve best practice? Clin Infect Dis. 2023;76:e864–6. 10.1093/cid/ciac728. 36056897 10.1093/cid/ciac728 PMC10169404

[130] Huang R, Xu R, Shi J, Yang Z, Zheng J, Wei D. Artesunate induces ferroptosis in osteosarcoma through NCOA4 -mediated ferritinophagy. FASEB J. 2025;39. 10.1096/fj.202403160R. 10.1096/fj.202403160R PMC11960798 40168090

[131] Jain S, Rana M, Angmo N, Vimal N. The paradox of heme oxygenase 1: from cellular defense to a tug of war between cancer promotion and prevention. Adv Biol. 2025;9. 10.1002/adbi.202500390. 10.1002/adbi.202500390 41236128

[132] Attucks OC, Jasmer KJ, Hannink M, Kassis J, Zhong Z, Gupta S, et al. Induction of heme oxygenase I (HMOX1) by HPP-4382: a novel modulator of Bach1 activity. PLoS ONE. 2014;9:e101044 10.1371/journal.pone.0101044. Jul 14. 25019514 10.1371/journal.pone.0101044 PMC4096395

[133] Eadie AL, Simpson JA, Brunt KR Teaching an old drug new tricks: Regulatory insights for the repurposing of hemin in cardiovascular disease. Pharmacol Res Perspect. 2024;12. 10.1002/prp2.1225. 10.1002/prp2.1225 PMC11194834 38923404

[134] Lorendeau D, Dury L, Genoux-Bastide E, Lecerf-Schmidt F, Simões-Pires C, Carrupt PA, et al. Collateral sensitivity of resistant MRP1-overexpressing cells to flavonoids and derivatives through GSH efflux. Biochem Pharm. 2014;90:235–45. 10.1016/j.bcp.2014.05.017. 24875445 10.1016/j.bcp.2014.05.017

[135] Wesołowska O, Mosiądz D, Motohashi N, Kawase M, Michalak K. Phenothiazine maleates stimulate MRP1 transport activity in human erythrocytes. Biochim. Biophys. Acta (BBA) Biomembr. 2005;1720:52–8. 10.1016/j.bbamem.2005.11.011. 10.1016/j.bbamem.2005.11.011 16364236

[136] Perloff MD, Von Moltke LL, Marchand JE, Greenblatt DJ. Ritonavir induces P-glycoprotein expression, multidrug resistance-associated protein (MRP1) expression, and drug transporter-mediated activity in a human intestinal cell line. J Pharm Sci. 2001;90:1829–37. 10.1002/jps.1133. 11745741 10.1002/jps.1133

[137] Brandmann M, Tulpule K, Schmidt MM, Dringen R. The antiretroviral protease inhibitors indinavir and nelfinavir stimulate Mrp1-mediated GSH export from cultured brain astrocytes. J Neurochem. 2012;120:78–92. 10.1111/j.1471-4159.2011.07544.x. 22017299 10.1111/j.1471-4159.2011.07544.x

[138] Schmitt SM, Stefan K, Wiese M. Pyrrolopyrimidine derivatives and purine analogs as novel activators of Multidrug Resistance-associated Protein 1 (MRP1, ABCC1). Biochim. Biophys. Acta (BBA) Biomembr. 2017;1859:69–79. 10.1016/j.bbamem.2016.10.017. 10.1016/j.bbamem.2016.10.017 27810353

[139] Cescon DW, She D, Sakashita S, Zhu CQ, Pintilie M, Shepherd FA, et al. NRF2 pathway activation and adjuvant chemotherapy benefit in lung squamous cell carcinoma. Clin Cancer Res. 2015;21:2499–505. 10.1158/1078-0432.CCR-14-2206. 25739673 10.1158/1078-0432.CCR-14-2206

[140] Nakamura T, Conrad M, Nakamura T, Conrad M. Exploiting ferroptosis vulnerabilities in cancer. Nat Cell Biol. 2024;26:1407–19. 10.1038/s41556-024-01425-8. 38858502 10.1038/s41556-024-01425-8

[141] Bottoni L, Minetti A, Realini G, Pio E, Giustarini D, Rossi R, et al. NRF2 activation by cysteine as a survival mechanism for triple-negative breast cancer cells. Oncogene. 2024;43:1701–13. 10.1038/s41388-024-03025-0. 38600165 10.1038/s41388-024-03025-0 PMC11136656

[142] Kerins MJ, Ooi A. A catalogue of somatic NRF2 gain-of-function mutations in cancer. Sci Rep. 2018;8:12846. 10.1038/s41598-018-31281-0. 30150714 10.1038/s41598-018-31281-0 PMC6110754

[143] Kim JW, Min DW, Kim D, Kim J, Kim MJ, Lim H, et al. GPX4 overexpressed non-small cell lung cancer cells are sensitive to RSL3-induced ferroptosis. Sci Rep. 2023;13:8872. 10.1038/s41598-023-35978-9. 37258589 10.1038/s41598-023-35978-9 PMC10232506

[144] Park JY, Kim YW, Park YK. Nrf2 expression is associated with poor outcome in osteosarcoma. Pathology. 2012;44:617–21. 10.1097/PAT.0b013e328359d54b. 23172081 10.1097/PAT.0b013e328359d54b

[145] Lister A, Nedjadi T, Kitteringham NR, Campbell F, Costello E, Lloyd B, et al. Nrf2 is overexpressed in pancreatic cancer: implications for cell proliferation and therapy. Mol Cancer. 2011;10:37. 10.1186/1476-4598-10-37. 21489257 10.1186/1476-4598-10-37 PMC3098205

[146] Müller F, Lim JKM, Bebber CM, Seidel E, Tishina S, Dahlhaus A, et al. Elevated FSP1 protects KRAS-mutated cells from ferroptosis during tumor initiation. Cell Death Differ. 2023;30:442–56. 10.1038/s41418-022-01096-8. 36443441 10.1038/s41418-022-01096-8 PMC9950476

[147] Yanagawa T, Omura K, Harada H, Nakaso K, Iwasa S, Koyama Y, et al. Heme oxygenase-1 expression predicts cervical lymph node metastasis of tongue squamous cell carcinomas. Oral Oncol. 2004;40:21–7. 10.1016/S1368-8375(03)00128-3. 14662411 10.1016/s1368-8375(03)00128-3

